# Double versus standard dose of tamsulosin with solifenacin for management of ureteral stent related symptoms

**DOI:** 10.1080/20905998.2025.2561225

**Published:** 2025-09-23

**Authors:** Ahmad Hefnawy, Mohamed Ramadan, Hisham Arafa, Tarek Osman, Mohamed Saied

**Affiliations:** Urology department, Ain Shams University, Cairo, Egypt

**Keywords:** Tamsulosin, solifenacin, stent-related symptoms

## Abstract

**Background:**

Ureteral stents are commonly used to ensure adequate drainage of the upper urinary tract in the presence, or anticipated occurrence, of ureteral obstruction. However, stent-related symptoms are frequent and represent a significant source of morbidity. Although several pharmacological treatment options are currently available, symptom relief remains often incomplete. In an effort to achieve better control of stent-related symptoms, we evaluated the efficacy of dose doubling in two commonly used drug combinations that have demonstrated symptomatic benefits in this context.

**Objective:**

To evaluate the efficacy of a double dose of solifenacin and tamsulosin compared to the standard dose of the same agents in alleviating stent-related symptoms.

**Patients and methods:**

A randomized clinical trial was conducted involving 64 Egyptian patients recruited from the outpatient clinic and inpatient services of the Urology Department at Ain Shams University Hospital over a six-month period.

**Results:**

After two weeks of treatment, the double dose of tamsulosin and solifenacin was significantly more effective in reducing stent-related symptoms compared to the standard dose. A statistically significant difference in symptom scores was observed between the double-dose and standard-dose groups. Side effects were more frequent in the double-dose group, with retrograde ejaculation and dry mouth occurring at significantly higher rates.

**Conclusion:**

The combination of tamsulosin and solifenacin is effective in alleviating stent-related symptoms. In patients who continue to experience symptoms despite standard-dose therapy, doubling the dose of both agents may offer additional symptomatic relief, albeit with a higher incidence of temporary and manageable side effects.

## Introduction

Ureteral stents were first described in 1967 and have since become a routine urologic procedure [1]. However, a considerable proportion of patients experience significant stent-related symptoms. Previous studies have reported the incidence of these symptoms as follows: frequency (50–60%), urgency (57–60%), dysuria (40%), incomplete emptying (76%), flank pain (19–32%), suprapubic pain (30%), and hematuria (25%) [2–5]. Joshi et al. demonstrated that stent-related urinary symptoms and pain result in a reduced quality of life in up to 80% of patients [6].

Several meta-analyses and systematic reviews have demonstrated significant improvements in stent-related symptoms following treatment with alpha-blockers, antimuscarinics, phosphodiesterase type 5 inhibitors (PDE5Is), and various combinations of antimuscarinics and alpha-blockers. However, despite these symptomatic improvements, none of the treatment approaches achieved complete resolution, with residual symptoms persisting across multiple domains of the Ureteral Stent Symptom Questionnaire (USSQ) [2,7,8].

Dose escalation has been employed to improve lower urinary tract symptoms (LUTS) in various clinical settings [9]. One study demonstrated additional symptomatic improvement with the use of a double dose of alpha-blockers for LUTS secondary to benign prostatic hyperplasia (BPH). Similarly [10], dose escalation of solifenacin from 5 mg to 10 mg resulted in better symptomatic improvement in patients with overactive bladder (OAB). Following a similar rationale, we aimed to evaluate this strategy for the management of LUTS associated with ureteral stents. Given that the combination of tamsulosin and solifenacin has demonstrated superior symptomatic benefit compared to placebo and monotherapy in multiple trials [2], this study was designed to assess the effect of standard-dose versus double-dose combination therapy on stent-related LUTS.

## Aim of the work

This pilot study aims to evaluate the effectiveness of a double dose versus a standard dose of tamsulosin combined with solifenacin in relieving stent-related symptoms.

## Patients and methods

### Study design

This study was a randomized clinical trial conducted on 64 Egyptian patients from the outpatient clinic at Ain Shams University Hospital over a six-month period from March 2024 to September 2024. The study population was randomized into 2 groups. Group A included 32 patients with stent related symptoms receiving double doses of tamsulosin (0.4 mg 2 capsules taken together per day) and Solifenacin 10 mg (10 mg tab taken once daily). Group B included 32 patients with stent related symptoms receiving a standard dose of tamsulosin (0.4 mg once daily) and solifenacin (5 mg once daily).

### Study procedure

Since this is a pilot study with no prior similar studies, we used an arbitrary sample size of 64 patients. Patient recruitment followed procedures that included stent placement, patients are routinely asked to come to the outpatient clinic after 1 week for routine post-operative assessment. Those reporting stent related symptoms were identified. In addition to their routine follow-up care they were offered participation in the study. A thorough discussion of the study including the risks and benefits was conducted. Interested patients signed an informed consent. Patients who agreed to participate in the study were randomly assigned to one of two groups (double dose or standard dose) using a simple random number table. Each patient was given a number in order of recruitment, and the number was matched with the random table to decide their group. At the initial visit, a baseline USSQ questionnaire [11] (which is considered as one important patient reported outcome measures PROMs for stent symptoms [12]) was administered. Patients then received medical treatment according to their assigned groups. A single-blind design was employed, wherein patients were unaware whether they were receiving the standard or double-dose treatment. Patients received medications in similar white unlabelled containers. After two weeks, all patients were re-evaluated through an interview, medication compliance was evaluated by pill count and an interview, and the USSQ questionnaire was re-administered to assess changes from baseline scores. The percentage of improvement in USSQ scores and rate of side effects were recorded and compared between the 2 groups. The primary outcome in this study was the percentage of improvement in the total USSQ score.

### Ethical considerations

Informed written consent was obtained from all participants. Patient confidentiality was maintained, and no identifying information was included in any publications. Confidential patient information was preserved on password protected hard drive by the principal investigator. No identifying patient information was retained after the final analysis was complete. The study was approved by the Research ethics committee of Faculty of medicine at Ain Shams University with IRB approval number of FMASU MS23/2023. The study was registered in clinicaltrials.gov with the registration number of NCT06955533. No funding was received for this study and there was no conflict of interest to be declared.

### Study population

**Figure 1.**
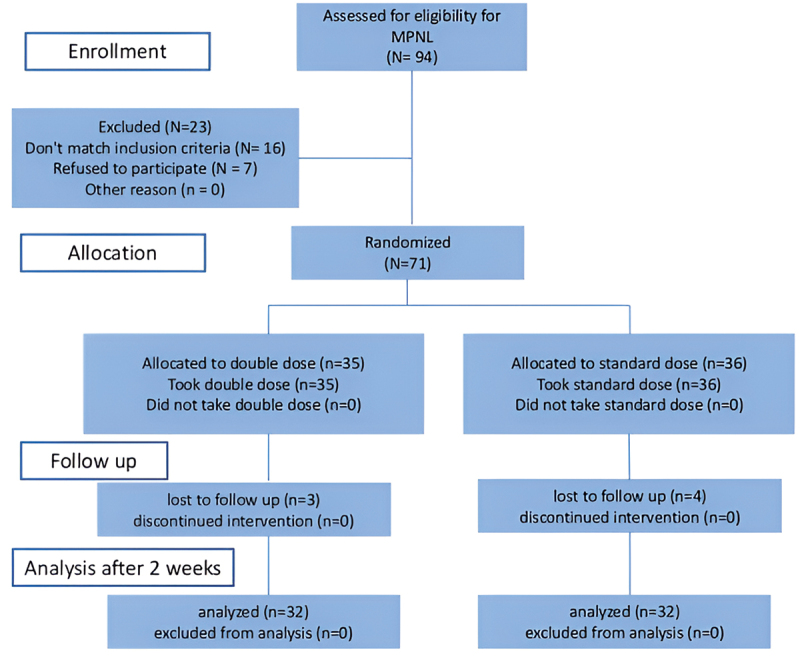
Consort flow chart.

We included patients older than 18 years of age, with presence of a polyurethane double-J (DJ) stent without pull strings in place, suffering from lower urinary tract symptoms (LUTS). Patients younger than 18 years of age, patients with a history of prostate or bladder surgery, lower urinary tract procedures, cancer, neurological conditions, pelvic radiation, diabetes, kidney dysfunction (acute or chronic), a solitary kidney, congenital urinary anomalies, or those taking medications such as α-blockers, beta-blockers, calcium channel blockers, 5-alpha reductase inhibitors, PDE5 inhibitors, anticholinergics, or nitrates were excluded. Additionally, patients with cardiac issues, residual stone fragments after surgery, multiple or bilateral ureteral stones, patients having/requiring long-term or bilateral stents, patients having stents with pull strings, patients requiring frequent stent changes, interstitial cystitis, chronic cystitis, prostatitis, pregnant or breastfeeding women, and those unavailable for follow-up were also excluded from the study (Figure 1).

### Statistical analysis

The data was analyzed using the Statistical Package for the Social Sciences (SPSS Inc., Chicago, IL, USA). Quantitative data was expressed as mean ± standard deviation and analyzed using the Student’s t-test. Non-parametric data was analyzed using Mann-Whitney test and expressed as median and interquartile range (IQR). Categorical data was represented as percentages and evaluated using the Chi-square test. A p-value of less than 0.05 was deemed statistically significant.

## Results

A total of 64 patients were recruited for the study, group A received double dose of tamsulosin and solifenacin, while group B received the standard dose of both medications. The two groups were well-matched at baseline, with no significant differences in any domain. Table 1 summarizes the baseline characteristics of the 2 groups. No patients were missed to follow-up or prematurely withdrawn from the study. Medication compliance was assessed in the 2 groups at the end of the follow-up period and was found to be 100% in the 2 groups. Regarding the treatment Efficacy, the double-dose group (group A) showed significantly greater improvements in urinary symptoms, body pain, general health, work performance, and total USSQ score compared to the standard-dose group (group B). Table 2 demonstrates the symptoms and degree of symptomatic improvement in each group. Side effects were more frequently observed in the double-dose group, however they only reached level of statistical significance in terms of retrograde ejaculation and dry mouth. This was summarized in Table 3. Procedure distribution was summarized in Table 4.

**Table 1.** Baseline patient characteristics.

Characteristic		Double Dose Group A (n=32)	Standard Dose Group B (n=32)	Test-value	P-value
Age		49.13 ± 9.02	43.75 ± 14.83	1.751	0.085
Gender	Male	30 (93.7%)	26 (81.25%)	2.286	0.131
	Female	2(6.3%)	6 (18.75%)		
Urinary Symptoms		34.00 ± 3.8	32.84 ± 3.6	1.23	0.215
Body Pain		15.44 ± 2.1	15.12 ± 2.4	0.61	0.540
General Health		14.24 ± 1.8	13.64 ± 3.4	0.85	0.401
Work Performance		12.65 ± 2.7	13.82 ± 2.4	−1.80	0.071
Sexual Matter		5.20 ± 0.6	5.55 ± 1.0	−1.52	0.13
Total USSQ Score		89.5 (83–96)	84 (80.5–87)	−1.837	0.066

Continuous variables were evaluated by t-test and represented as mean and standard deviation.

Gender comparison used Chi-Square test.

Total USSQ was represented as median and IQR (Mann-Whitney test).

**Table 2.** Comparison of outcomes between double dose and standard dose groups after treatment.

Domain	Double Dose Group A (*n* = 32)	Standard Dose Group B (*n* = 32)	P-value	% Improvement (Double vs. Standard) ± SD (C.I) P-value
Urinary Symptoms	18.4 ± 2.5	22.10 ± 3.8	0.0001	−46.87%±2.88(−47.86 – −45.87) vs. −27.85%±3.89 (−29.19 – −26.5) P-value 0.0001
Body Pain	10.35 ± 1.5	11.56 ± 1.9	0.006	−32.24%±9.68 (−35.59 – −28.88) vs. −23.98%±5.57(−25.91 – −22.05) P-value 0.0001
General Health	10.18 ± 1.45	10.72 ± 0.88	0.077	−30.0%±9.99 (−33.46 – −26.53) vs. −23.0%±15.62 (−28.41 – −17.58) P-value 0.037
Work Performance	10.60 ± 2.2	11.85 ± 2.5	0.0377	−17.42%±13.79 (−22.2 – −12.64) vs. −15.37%±15.72(−20.82 – −9.92) P-value 0.58
Sexual Matter	5.13 ± 0.34	4.87 ± 0.61	0.045	0(0–0) vs.0(−26.67–0) * P-value 0.116
Total USSQ Score	60 (54.5–63)	63 (60.5–70)	0.001	−32.29%±6.64 (−34.591 – −29.989) vs. −25.0%±5.6 (−26.940 – −23.060) P-value 0.0001

Percentages of improvement are represented as mean, SD and confidence intervals. Sexual matter is represented as median with IQR (Mann Whitney test).

Mean percentage of improvement was calculated by summing the percentage of improvement of each patient and obtaining the average (average of percentage of improvement) for each group.

**Table 3.** Side effects comparison.

Side Effect	Double Dose Group A (n = 32)	Standard Dose Group B (n = 32)	P-value
Retrograde Ejaculation	8 (25.0%)	2 (6.3%)	0.039
Dry Mouth	10 (31.3%)	3 (9.4%)	0.030
Fatigue	7 (21.9%)	4 (12.5%)	0.320
Dizziness	6 (18.8%)	3 (9.4%)	0.474
Orthostatic Hypotension	5 (15.6%)	3 (9.4%)	0.708
Constipation	6 (18.8%)	2 (6.3%)	0.257
Blurring of Vision	5 (15.6%)	4 (12.5%)	0.719

Chi-Square test.

**Table 4.** Distribution of procedures among the 2 study groups.

	Double Dose (n = 32) Group A	Standard Dose (n = 32) Group B	P value
Semirigid ureteroscopy	24	22	0.834
Retrograde intrarenal surgery	6	7	
PCNL	2	3	

Chi-Square test.

A post-hoc power analysis was performed for the primary outcome (percentage of improvement of the total USSQ score) demonstrated a power of 99.6% to detect the demonstrated effect size of 1.18 using an independent sample t-test with the alpha value set at 5%.(calculated using G*power software v3.1.9.2) [13].

## Discussion

Ureteral stents are widely utilized in urology to maintain upper urinary tract drainage or to support ureteral anastomoses. However, the exact pathophysiological mechanisms underlying stent-related symptoms (SRSs) remain poorly understood [14].

Ongoing research continues to explore the effectiveness of various therapeutic protocols, utilizing different pharmacological agents, to alleviate symptoms associated with ureteral stents. A meta-analysis conducted by Jian et al. (2019) demonstrated that the combination of tamsulosin 0.4 mg and solifenacin 5 mg may represent the most effective intervention for stent-related symptoms (SRSs) [15]. Additionally, a study by Abelaal et al. reported that combined therapy with tamsulosin 0.4 mg daily and solifenacin 5 mg daily is a safe, well-tolerated, and more effective approach for managing stent-related symptoms compared to monotherapy with either agent [16].

Other pharmacological options have also been explored, among the studies investigating pharmacological interventions for stent-related symptoms, Shalaby et al. evaluated the use of a higher dose of solifenacin (10 mg). Conversely tamsulosin 0.8 mg has been only studied in the context of benign prostatic hyperplasia treatment (BPH) [17,18], its effectiveness in managing lower urinary tract symptoms (LUTS) specifically associated with ureteral stents has not been previously explored.

Our study was the first to evaluate a combination of tamsulosin 0.8 mg and solifenacin 10 mg in the context of stent related symptoms. To explore potential for additional symptomatic improvements with dose escalation, we evaluated the improvement with this treatment regimen against the combination therapy (0.4 mg Tamsulosin and 5 mg Solifenacin) that has been successful in multiple trials [8,15,16].

All preoperative data and baseline Characteristics in the two treatment groups were well-matched at baseline, with no significant differences in any domain. Both groups in our study demonstrated similar baseline USSQ scores across all domains before treatment. However, after two weeks, (Group A) patients experienced significantly better symptom relief than those in (Group B), Where the USSQ sub-score became significantly lower in (Group A) in almost all domains except for sexual matters and work performance.

In (Group A), urinary symptoms score decreased by 46.87% with only 27.85% in (Group B), body pain score decreased by 32.24% in (Group A) and 23.98% in (Group B). General health score decreased by 30% in (Group A) and 23% in (Group B). The total USSQ score decreased by 32.29% in (Group A) with only 25% in (Group B). All differences were found to be statistically significant. Given the pilot nature of this study, we were not able to perform clear comparisons between the findings in our study with similar previous studies regarding the differing efficacy of both treatment regimens. However, limited correlations can be made between the findings in (Group B) and other studies.

The results of our study differ from those reported by Abelaal et al., who observed a 30.8% improvement in the USSQ total score with tamsulosin monotherapy and a 46.7% improvement with combination therapy. The lower improvement rates in our study may be attributed to differences in study design, since abdelaal et al. was a multicenter study with significant patient heterogenicity and had a significantly longer follow up period allowing for the full medication effect to take place [16].

Regarding side effects, fatigue, dizziness, retrograde ejaculation, orthostatic hypotension, dry mouth, and constipation were more commonly reported in the double-dose group. However, significant differences were observed only in retrograde ejaculation and dry mouth with (p-value of 0.039, 0.030 respectively), however there was no patient withdrawal from the study because of the increased incidence of side effects.

Unlike our study, Abdelhamid et al. (2017) assessed two doses of solifenacin succinate (5 mg and 10 mg) for stent-related symptoms, finding no significant differences between doses. Reported side effects, such as headache, dry mouth, and constipation, were mostly minor and self-limiting, likely differing from our results due to the use of combination therapy [19].

Aharony et al. explored the benefits of increasing tamsulosin dosages in patients with benign prostatic hyperplasia (BPH) and noted that adverse effects and treatment withdrawals increased with higher doses [20]. In line with our findings, Lepor et al. examined the efficacy and safety of two tamsulosin doses (0.4 mg/day and 0.8 mg/day) for BPH. Their study found a higher discontinuation rate in the 0.8-mg/day group (13%) compared to the 0.4 mg/day and placebo groups (7% and 9%, respectively), attributed to increased adverse events like abnormal ejaculation, dizziness, chest pain, and hypotension [21] .

The higher discontinuation rates with increased tamsulosin doses in these studies may be due to the prolonged treatment duration for BPH, whereas our study’s two-week period did not involve any treatment discontinuation.

To our knowledge this is the first study to evaluate double dose tamsulosin and solifenacin in improving stent related symptoms, considering our findings it would be interesting to study safety and efficacy on a larger population and for a longer duration. The limitations of our study include a relatively small sample size and short follow up duration that might have not enabled us to capture the full side effect profile with medication change.Additionally our study groups were heterogenous, as participants underwent different procedures requiring stent placement for varying durations. Nevertheless, these procedures were evenly distributed between the study groups, minimizing potential bias.

## Conclusion

Our study found that a double dose of tamsulosin and solifenacin provided better symptomatic improvement than the standard dose of the same combination but with a modest increase in side effects. Further larger-scale studies are needed to affirm the findings and to provide clear recommendations.

## Abbreviations list

LUTS: Lower urinary tract symptoms
OAB: Overactive bladder
BPH: Benign prostatic hyperplasia
DJ: Double J
PDE5: Phosphodiesterase 5
SPSS: Statistical package for social sciences
USSQ: Ureteral stent symptom questionnaire
SRSs: stent related symptoms

## References

[1] Zimskind PD, Fetter TR, Wilkerson JL. Clinical use of long-term indwelling silicone rubber ureteral splints inserted cystoscopically. J Urol. 1967 May;97(5):840–844. doi: 10.1016/S0022-5347(17)63130-6 6025928

[2] Zhou L, Cai X, Li H, et al. Effects of α-blockers, antimuscarinics, or combination therapy in relieving ureteral stent-related symptoms: a meta-analysis. J Endourol. 2015 Jun 1;29(6):650–656. doi: 10.1089/end.2014.0715 25491604 PMC4490592

[3] Haleblian G, Kijvikai K, De La Rosette J, et al. Ureteral stenting and urinary stone management: a systematic review. J Urol. 2008 Feb 1;179(2):424–430. doi: 10.1016/j.juro.2007.09.026 18076928

[4] Joshi HB, Okeke A, Newns N, et al. Characterization of urinary symptoms in patients with ureteral stents. Urology. 2002 Apr 1;59(4):511–516. doi: 10.1016/S0090-4295(01)01644-2 11927301

[5] Thomas RA. Indwelling ureteral stents: impact of material and shape on patient comfort. J Endourol. 1993 Apr;7(2):137–140. doi: 10.1089/end.1993.7.137 8518826

[6] Joshi HB, Stainthorpe A, MacDonagh RP, et al. Indwelling ureteral stents: evaluation of symptoms, quality of life and utility. J Urol. 2003 Mar;169(3):1065–1069. doi: 10.1097/01.ju.0000048980.33855.90 12576847

[7] Betschart P, Zumstein V, Piller A, et al. Prevention and treatment of symptoms associated with indwelling ureteral stents: a systematic review. Int J Urol. 2017 Apr;24(4):250–259. doi: 10.1111/iju.13311 28236323

[8] Pecoraro A, Peretti D, Tian Z, et al. Treatment of ureteral stent-related symptoms. Urol Int. 2023 Mar 22;107(3):288–303. doi: 10.1159/000518387 34818261

[9] Ahmed Hassaan AM, Raouf Salman BM, El Sayed Selim MA, et al. Single versus double dose tamsulosin for patients with moderate or severe lower urinary tract symptoms due to benign prostatic hyperplasia: prospective randomized study. Egypt J Hosp Med. 2024 Jan 1;94(1):300–304.

[10] Chapple CR, Cardozo L, Steers WD, et al. Solifenacin significantly improves all symptoms of overactive bladder syndrome. Int J Clin Pract. 2006 Aug;60(8):959–966. doi: 10.1111/j.1742-1241.2006.01067.x 16893438 PMC1619936

[11] Joshi HB, Newns N, Stainthorpe A, et al. Ureteral stent symptom questionnaire: development and validation of a multidimensional quality of life measure. J Urol. 2003 Mar;169(3):1060–1064. doi: 10.1097/01.ju.0000049198.53424.1d 12576846

[12] Mehmi A, Jones P, Somani BK. Current status and role of patient-reported outcome measures (PROMs) in endourology. Urology. 2021 Feb 1;148:26–31. doi: 10.1016/j.urology.2020.09.022 32991909

[13] Faul F, Erdfelder E, Lang A-G, et al. G*Power 3: a flexible statistical power analysis program for the social, behavioral, and biomedical sciences. Behav Res Methods. 2007;39(2):175–191. doi: 10.3758/BF03193146 17695343

[14] Koprowski C, Kim C, Modi PK, et al. Ureteral stent-associated pain: a review. J Endourol. 2016 Jul 1;30(7):744–753. doi: 10.1089/end.2016.0129 27125392

[15] Jian Z, Chen Y, Liu Q, et al. Combination of solifenacin and tamsulosin may provide additional beneficial effects for ureteral stent-related symptoms—outcomes from a network meta-analysis. World J Urol. 2019 Feb 12;37(2):289–297. doi: 10.1007/s00345-018-2404-6 30030658

[16] Abdelaal AM, Al-Adl AM, Abdelbaki SA, et al. Efficacy and safety of tamsulosin oral-controlled absorption system, solifenacin, and combined therapy for the management of ureteric stent-related symptoms. Arab J Urol. 2016 Jun 1;14(2):115–122. doi: 10.1016/j.aju.2016.01.004 27489738 PMC4963155

[17] Osman T, Elawady H, Fawaz K, et al. Evaluation of tamsulosin 0.4 mg versus 0.8 mg in management of lower urinary tract symptoms due to benign prostatic enlargement. Int Urol Nephrol. 2024 Jun;56(6):1811–1816. doi: 10.1007/s11255-023-03912-7 38219259 PMC11090953

[18] Dogha MM, Shaker H, Abdelazeez A, et al. Tamsulosin 0.8 mg daily dose in management of BPH patients with failed tamsulosin 0.4 mg monotherapy and unfit for surgical intervention. World J Urol. 2024 Jun 1;42(1):365. doi: 10.1007/s00345-024-05050-w 38822877 PMC11144140

[19] Abdelhamid MH, Zayed AS, Ghoneima WE, et al. Randomized, double-blind, placebo-controlled trial to compare solifenacin versus trospium chloride in the relief of double-J stent-related symptoms. World J Urol. 2017 Aug;35(8):1261–1268. doi: 10.1007/s00345-016-1988-y 28050642

[20] Aharony S, Lam O, Corcos J. Is there a demonstrated advantage to increase tamsulosin dosage in patients with benign prostatic hyperplasia? Urology. 2014 Aug 1;84(2):493–494. doi: 10.1016/j.urology.2014.04.018 25065994

[21] Lepor H. Long-term evaluation of tamsulosin in benign prostatic hyperplasia: placebo-controlled, double-blind extension of phase III trial. Urology. 1998 Jun 1;51(6):901–906. doi: 10.1016/S0090-4295(98)00127-7 9609624

